# Comparison of the fertility of tumor suppressor gene-deficient C57BL/6 mouse strains reveals stable reproductive aging and novel pleiotropic gene

**DOI:** 10.1038/s41598-021-91342-9

**Published:** 2021-06-11

**Authors:** Masaoki Kohzaki, Akira Ootsuyama, Toshiyuki Umata, Ryuji Okazaki

**Affiliations:** 1 Department of Radiobiology and Hygiene Management, Institute of Industrial Ecological Sciences, University of Occupational and Environmental Health, Japan, 1-1 Iseigaoka Yahatanishi-ku, Kitakyushu, 807-8555 Japan; 2 Department of Radiation Biology and Health, School of Medicine, University of Occupational and Environmental Health, Japan, 1-1 Iseigaoka Yahatanishi-ku, Kitakyushu, 807-8555 Japan; 3 Radioisotope Research Center, Facility for Education and Research Support, University of Occupational and Environmental Health, Japan, 1-1 Iseigaoka Yahatanishi-ku, Kitakyushu, 807-8555 Japan

**Keywords:** Cancer, Developmental biology, Genetics, Physiology, Diseases, Medical research, Oncology, Risk factors

## Abstract

Tumor suppressor genes are involved in maintaining genome integrity during reproduction (e.g., meiosis). Thus, deleterious alleles in tumor suppressor-deficient mice would exhibit higher mortality during the perinatal period. A recent aging model proposes that perinatal mortality and age-related deleterious changes might define lifespan. This study aimed to quantitatively understand the relationship between reproduction and lifespan using three established tumor suppressor gene (p53, APC, and RECQL4)-deficient mouse strains with the same C57BL/6 background. Transgenic mice delivered slightly reduced numbers of 1st pups than wild-type mice [ratio: 0.81–0.93 (*p* = 0.1–0.61)] during a similar delivery period, which suggest that the tumor suppressor gene-deficient mice undergo relatively stable reproduction. However, the transgenic 1st pups died within a few days after birth, resulting in a further reduction in litter size at 3 weeks after delivery compared with that of wild-type mice [ratio: 0.35–0.68 (*p* = 0.034–0.24)] without sex differences, although the lifespan was variable. Unexpectedly, the significance of reproductive reduction in transgenic mice was decreased at the 2nd or later delivery. Because mice are easily affected by environmental factors, our data underscore the importance of defining reproductive ability through experiments on aging-related reproduction that can reveal a trade-off between fecundity and aging and identify RECQL4 as a novel pleiotropic gene.

## Introduction

**Figure 1:**
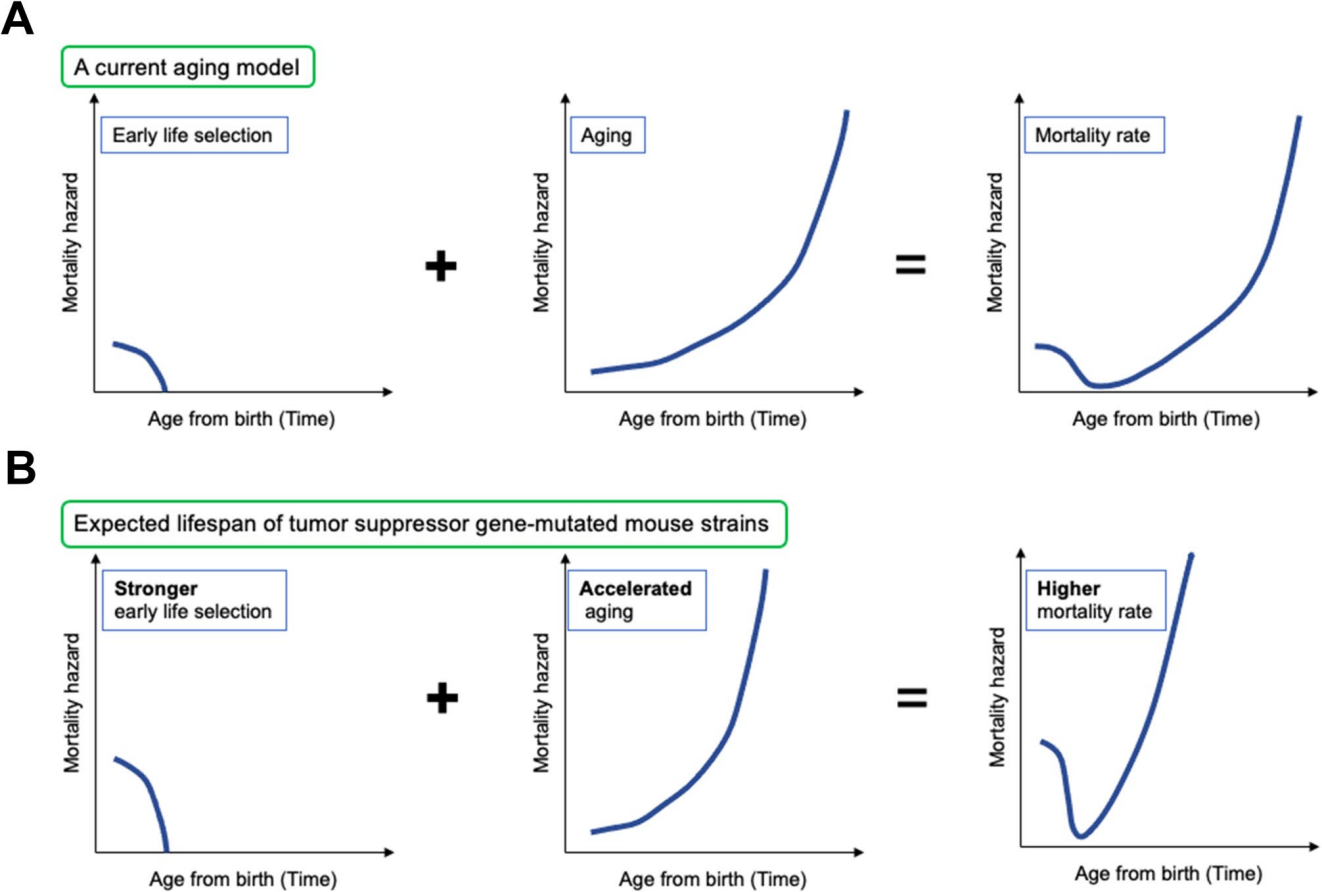
A current aging model and expected outcomes in tumor suppressor gene-deficient mice. (**A**) A simple aging model considers only aging effects (middle) such as epigenetic changes and genetic alterations, whereas a current aging model proposed by Dr. Gladyshev’s group^9^ also considers the effect of early life selection (left) on lifespan (right). (**B**) If the absence of tumor suppressor gene is associated with less genome integrity protection, we expect accelerated aging (middle) associated with stronger early life selection (left), which results in a markedly shorter lifespan (right).

Aging is a convoluted process that is consistent with the multiplicity aspect of biology, and several aging hypotheses have been proposed^1^. The “mutation accumulation” hypothesis proposed by Dr. Medawar estimates that late-acting deleterious germline mutations exhibit only a slight influence after maturity as the force of natural selection declines with age^2^. Later, Dr. Williams proposed the “antagonistic pleiotropy” hypothesis, which postulates that the aging process is influenced by pleiotropic genes that exert beneficial effects on reproduction in early life when natural selection is strong, but deleterious effects in later life when natural selection is weak^3^. These hypotheses imply that accumulated mutations can be evolutionarily inherited by the offspring. Indeed, considerable numbers of mutations are transmitted to the next generation in humans^4^. In contrast, limited evidence is available for antagonistically pleiotropic genes with the exception of p53, which plays a role in the trade-off between aging and cancer^5,6^. Therefore, the “disposable soma” hypothesis was developed by Dr. Kirkwood^7^ to explain how longevity is controlled by some genes that regulate somatic maintenance and repair functions in a stochastic and plastic manner. This theory can explain the accumulation rate of multiple types of damage in complex networks with maintenance and repair functions^1^. In this regard, long-lived animals, including humans, should minimize the damage accumulated by tumor suppressor genes and decrease the risk of malignancies that shorten life expectancy^8^. An aging model (Fig. 1A), which states that aging starts very early in life, overall mortality is the sum of the accumulation of genetic/epigenetic damage with age, and early life mortality plays a role in negative selection against parental deleterious alleles, was recently proposed^9^. If genome instability is promoted in tumor suppressor gene-deficient mice, both higher perinatal mortality and more accumulated damage should shorten the lifespan (Fig. 1B). In other words, if the lifespan of tumor suppressor gene-deficient mice is unchanged, lower perinatal mortality and/or decreased age-related accumulation of deleterious damage should theoretically occur.

Genetically altered mice are susceptible to infertility for several reasons, including endocrinopathies, genomic instability associated with germline function, and defects in hormone control^10^. Germline meiosis is mediated by genome recombination, and hence, infertility or subfertility is observed in transgenic mice lacking DNA repair genes such as ataxia telangiectasia mutated (ATM), mismatch repair (MMR) genes, retinoblastoma gene (RB)^11^, p53^12–14^, and RECQ helicase genes^15^. Five RECQ helicases, RECQL1, BLM, WRN, RECQL4 and RECQL5, are involved in genome integrity and are conserved in vertebrates^16^. Defects in BLM, WRN, and RECQL4 cause hereditary disease syndromes and cancers. Compared with other RECQ helicases, RECQL4 has been poorly characterized because N-terminal the targeted knockout of RECQL4 is lethal^16^. Therefore, viable RECQL4 knockout mice have been generated only by targeting the sequence encoding the helicase domain (HD) in the middle of the RECQL4 gene^17^. Germline mutations in RECQL4 cause the hereditary cancer predisposition syndrome Rothmund–Thomson syndrome (RTS) type II^18,19^ in addition to Baller–Gerold syndrome (BGS) and RAPADILINO syndrome^20^. Curiously, the lifespan of patients with RTS is not shortened if they do not also have cancer^21^, and this normal lifespan has also been observed in RECQL4-deficient mice^17^. Ninety-five percent of patients with familial adenomatous polyposis (FAP) harboring adenomatous polyposis coli (APC) gene mutations develop adenomas by 35 years of age and eventually develop colorectal cancer by age 40–50^22^. APC^Min/+^ mice have been used as a model of FAP for studying intestinal cancer for three decades^23^, and the APC gene is a well-defined tumor suppressor gene. Notably, the combination of the APC^Min^ allele with BLM, RECQL4, or RECQL5 deficiency results in increased tumor multiplicity in the small intestine^17,24,25^. Therefore, APC, BLM, RECQL4, and RECQL5 are considered tumor suppressor genes. In addition, p53 acts as a tumor suppressor in more advanced organisms, because somatic mutations in the p53 gene are the most frequently observed mutations in patients with cancer^14^. Heterogeneous germline mutations in the p53 gene are linked to Li-Fraumeni syndrome (LFS), and these patients are predisposed to develop a wide spectrum of cancers at younger ages (41% by age 18 years) than the general population^26^. Although p53, APC, and RECLQ4 are involved in genome stability^14,16,23^, mice with a completely nonfunctional p53 (i.e., p53 knockout) can develop normally with no apparent histopathological defects^27^. A sex-dominant defect associated with developmental abnormalities has been reported, as demonstrated by the finding of 23% fewer female p53^−/−^ mice than wild-type mice at weaning^12^. Female p53^−/−^ mice were reportedly exhibit marked reductions in their pregnancy rate (from 100 to 27%) and litter size (from 6.71 to 0.69 pups) induced by decreased leukemia inhibitory factor (LIF) expression^13^. In contrast, p53-mediated LIF regulation is not directly involved in reproductive decline, as suggested by Hirota et al*.*^28^. Moreover, homozygous p53 mutant individuals in a zebrafish model of LFS are completely viable and fertile^29^. Thus, the fertility of vertebrates in the absence of p53 remains to be determined.

**Figure 2:**
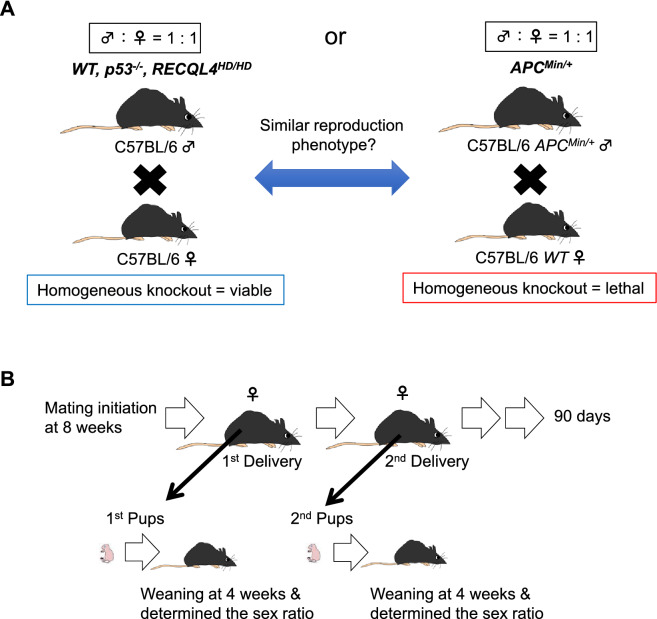
Overview of the experimental process for determining the reproductive ability of transgenic mice. Experimental procedure used for the examination of reproductive ability. (**A**) One-by-one pair mating experiment for understanding reproductive ability. APC^Min/Min^ mice exhibit embryonic lethality, and female APC^Min/+^ mice have a high likelihood of death within several months. Hence, male APC^Min/+^ mice and female wild-type mice were used for mating. (**B**) Timing of mating and weaning timing for examining reproductive ability over a definite period (90 days). See the details in the Methods.

We questioned why the mice deficient in the tumor suppressor gene RECQL4 and patients with RTS exhibit a normal lifespan despite showing increased genetic instability^17,21^. If a recent aging model is true, the early life mortality of RECQL4-deficient mice should be significantly improved. However, no such impression of improved early mortality has been observed in RECQL4-deficient mice. Therefore, we aimed to quantitatively understand the relationship between perinatal mortality and lifespan in three independent well-established tumor suppressor gene-deficient mouse strains. To achieve this purpose, we established an experimental procedure with the same environmental conditions to compare and determine the aging-associated fertility of p53^−/−^, APC^Min/+^, and RECQL4^HD/HD^ mice with a nearly identical C57BL/6 background (Materials and Methods). p53, APC, and RECQL4 are involved in distinct signaling pathways named the ATM-p53-p21 pathway^30^, the WNT signaling pathway^31^, and possibly the AKT-YB1-MDR1 pathway^32^, respectively. In addition, the APC gene is essential for viability, and APC^Min/+^ male mice should be mated with wild-type female mice to avoid embryonic lethality in the offspring. We compared the effect of the parental genotype in the selection against deleterious alleles for three independent tumor suppressor genes during development (Fig. 2A). Thus, these different transgenic mouse phenotypes might provide generalized information about reproduction in tumor suppressor gene-deficient mice. Importantly, our simple experiments with a relatively small number of mice had sufficient power to reveal not only the recovery of reproduction at the 2nd or later delivery in tumor suppressor gene-deficient mice but also the possibility of two different types of tumor suppressor genes with negative or positive effects on aging. The statistical power achieved with a relatively small number of mice in our simple experiments meets the requirement for ethical issues in animal research. Because reproductive ability can provide valuable information about transgenic mice, these experiments might contribute to further examination of the aging-related reproductive phenotypes of genetically modified mouse strains generated using newly established technology, including the CRISPR/Cas9 system.

## Results

### Establishment of an experiment for the assessment of age-related reproduction

Mice are characterized by high perinatal mortality, as observed in humans^9,33^. Perinatal mortality can be accelerated in transgenic mice due to chance failure, chromosomal instability, and harmful mutations. Indeed, we observed lower breeding in the transgenic mice than in wild-type C57BL/6 mice. Therefore, we maintained the transgenic mice by mating one to three male mice with two to four female mice (an equal or greater number of female mice) (Supplementary Fig. 1A). However, some female p53^−/−^ mice delivered many pups (Supplementary Fig. 1B; n > 7). These pups often died within a few days, and the dead pups rapidly disappeared by either parental cannibalism^33^ or rearing maintenance by our animal care facility. These findings imply that p53^−/−^ mice exhibit relatively normal embryonic implantation and litter sizes during the perinatal period, in contrast to the results of a previous study^13^. Therefore, we conducted an experiment to definitively characterize the reproductive ability of the mice by analyzing the survival rate of the pups at 3 weeks of age to distinguish between the perinatal period (when the high mortality is observed) and the puberty period (when minimal mortality is observed)^9^. We performed comparative experiments to assess the reproduction of three independent p53^−/−^, APC^Min/+^, and RECQL4^HD/HD^ mouse strains by setting the same age at weaning (4 weeks of age) and mating (8 weeks of age) for the mating pairs (Fig. 2B). p53 is considered to be the clearest example of an antagonistically pleiotropic gene^1^, and might serve as a reference for assessing the reproductive ability of tumor suppressor genes in mice. Assessing the perinatal mortality of RECQL4^HD/HD^ mice is important for understanding the relationship between perinatal mortality and age-related deleterious changes in RECQL4^HD/HD^ mice that show a normal lifespan^17^. To determine the decline in fecundity with aging, paired mice were placed in cages for at least 90 days, and reproductive ability data were obtained at the 1st delivery and subsequent deliveries (Fig. 2B).

### Tumor suppressor gene-deficient C57BL/6 mice showed relatively stable reproduction with aging compared with wild-type mice

**Figure 3:**
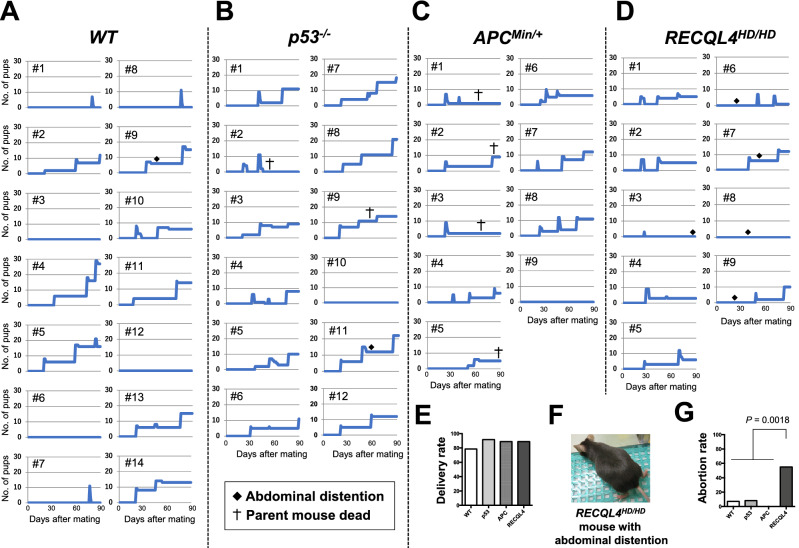
Cumulative pup numbers of each mating pair in the four strains within a 90-day period. Cumulative pup numbers produced by each mating pair of wild-type C57BL/6 N (**A**), p53^−/−^ (**B**), APC^Min/+^ (**C**), and RECQL4^HD/HD^ (**D**) mice. The diamonds indicate mice with abdominal distention. The crosses indicate the death of one parent mouse. (**E**) Delivery rates of the four mouse strains. (**F**) Representative image of a RECQL4^HD/HD^ mouse with abdominal distention. (**G**) Abortion rates of the four mouse strains. Ordinary one-way ANOVA for multiple comparisons among the four strains indicated a *p* value of 0.0018 between the RECQL4^HD/HD^ mice and the other mouse strains.

**Figure 4:**
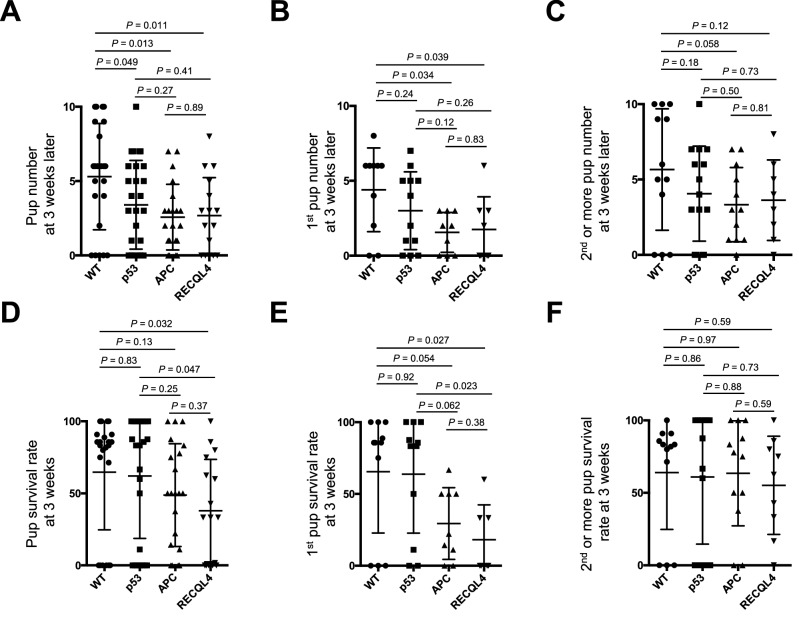
Pup numbers and survival rates at 3 weeks after birth. Pup numbers at 3 weeks after birth: total (**A**), in the 1st litter (**B**), and in the 2nd or later litter (**C**). The mean pup numbers are indicated by the bars. *p* values were determined by the Mann–Whitney U test or Welch’s t-test. Pup survival rates at 3 weeks after birth: total (**D**), in the 1st litter (**E**), and in the 2nd or later litter (**F**). The mean pup numbers are indicated by the bars. *p* values were determined by the Mann–Whitney U test or Welch’s t-test. p53: p53^−/−^, APC: APC^Min/+^, RECLQ4: RECQL4^HD/HD^.

Unexpectedly, these experiments showed that all the transgenic mouse strains exhibited a high delivery rate compared with that of wild-type C57BL/6 mice (Fig. 3A–E). Note that we assessed the delivery rate instead of the pregnancy rate in this study. Due to this setting, we noticed that RECQL4^HD/HD^ mice showed a higher abortion rate (Fig. 3F,G *p* = 0.0018; denoted by the diamonds in Fig. 3A–D). As expected, the pup numbers at 3 weeks obtained with the p53^−/−^, APC^Min/+^, and RECQL4^HD/HD^ mice were lower than those found with wild-type mice (Fig. 4A–C). In contrast, the p53^−/−^ pup survival rate 3 weeks after birth was higher than that of APC^Min/+^ or RECQL4^HD/HD^ pups in the 1st litter (Fig. 4D–F). Consistent with a previous report^34^, male APC^Min/+^ and wild-type female pairs showed reproductive impairment (Fig. 4), supporting the hypothesis that APC is needed for the integrity of spermatogenesis.

**Figure 5:**
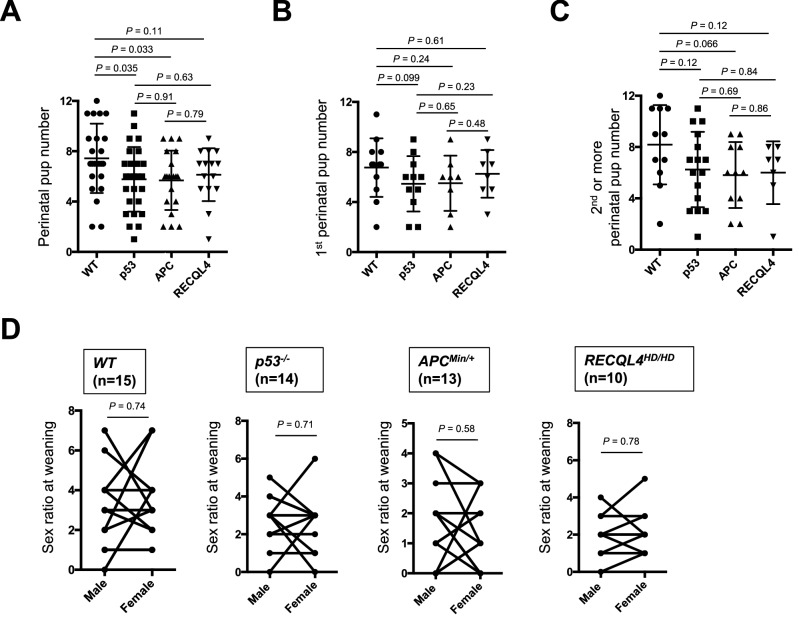
Pup numbers during the perinatal period and sex ratio results. Pup numbers during the perinatal period: total (**A**), in the 1st litter (**B**), and in the 2nd or later litter (**C**). The mean pup numbers are indicated by the bars. *p* values were determined by the Mann–Whitney U test or Welch’s t-test. (**D**) The sex ratio is shown as pairs at weaning. Each bar indicates the ratio of male and female pups from the same mother at weaning. *p* values were determined by the nonparametric Wilcoxon matched-pairs signed-rank test for the evaluation of sex bias. p53: p53^−/−^, APC: APC^Min/+^, RECLQ4: RECQL4^HD/HD^.

**Figure 6:**
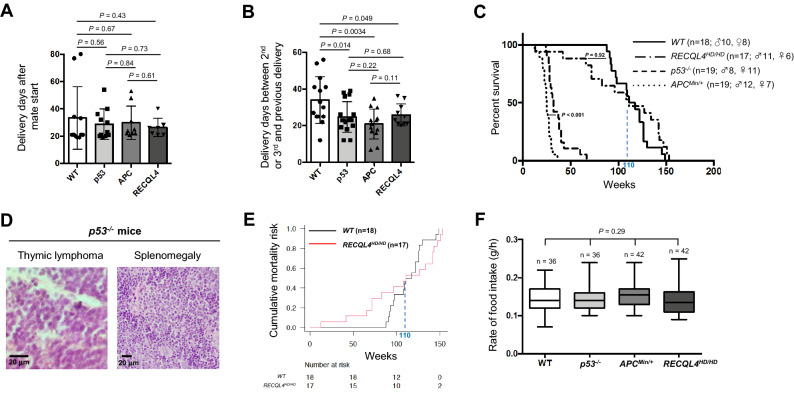
Comparative analysis of the delivery periods and lifespan among wild-type and tumor suppressor gene-deficient mice. Delivery periods between mating initiation and the 1st delivery (**A**) and between the 2nd or later delivery and the previous delivery (**B**). The mean number of days is indicated by the bars. *p* values were determined by the Mann–Whitney U test or Welch’s t-test. p53: p53^−/−^, APC: APC^Min/+^, RECLQ4: RECQL4^HD/HD^. (**C**) Kaplan–Meier survival curves showing the lifespan of the wild-type C57BL/6 J (n = 18; 10 male, 8 female), p53^−/−^(n = 19; 8 male, 11 female), APC^Min/+^ (n = 19; 12 male, 7 female), and RECQL4^HD/HD^ (n = 17; 11 male, 6 female) mouse strains. *p* values were determined by the Gehan-Breslow-Wilcoxon test. (**D**) Representative images of hematoxylin–eosin (H&E) staining of thymic lymphoma and splenomegaly in p53^−/−^ mice. Thymic lymphoma was indicated by a faint cytoplasm and convoluted nuclear contours, and splenomegaly was indicated by less-defined lymphoid follicles. (**E**) Cumulative mortality risk in wild-type mice and RECQL4^HD/HD^ mice. A Cox regression analysis was used to estimate the hazard ratios (95% confidential interval) of the age-dependent groups of RECQL4^HD/HD^ mice with those of wild-type mice, as shown in Table 1. (**F**) The rate of food intake (gram per hour) for the wild-type, p53^−/−^, APC^Min/+^, and RECQL4^HD/HD^ strains was calculated as described in Materials and Methods and is depicted in the box‐and‐whisker plot showing the medians of n > 35 samples, the first and third quartiles (boxes) and the overall ranges (whiskers). Ordinary one-way ANOVA for multiple comparisons among the four strains generated a *p* value of 0.29.

Because the numbers of p53^−/−^ pups were considerable (n > 7) (Supplementary Fig. 1B), we carefully analyzed the pup numbers just after delivery to assess the mortality rate during the perinatal period. The litter sizes of the p53^−/−^, APC^Min/+^, and RECQL4^HD/HD^ mice were slightly lower than those of the wild-type mice (Fig. 5A–C). A previous study demonstrated that p53^−/−^ mice gave birth to fewer female pups (~ 23%) than male pups^12^, and we revisited this sex bias in transgenic mice. To compare the sex ratio of the pups from the same mother mouse at weaning, we used a paired bar graph with each male and female pair of pups at weaning. As shown in Fig. 5D, no sex bias was observed with any examined mouse strain (*p* > 0.57). We then analyzed the durations of the 1st and subsequent delivery periods to examine whether aging-associated reproduction was affected. Strikingly, p53^−/−^, APC^Min/+^, and RECQL4^HD/HD^ mice showed delivery periods comparable to those of wild-type mice (Fig. 6A). In addition, when weaning was delayed for 2 weeks (until 6 weeks after birth), some p53^−/−^ and RECQL4^HD/HD^ female mice showed early sexual maturity (p53^−/−^ mice: born on 6 May, weaned on 19 June, gave birth on 1 July; RECQL4^HD/HD^ mice: born on 27 April, weaned on 8 June, gave birth on 1 July). These data suggest that reproduction associated with aging is not significantly affected in tumor suppressor gene-deficient mice.

### Wild-type C57BL/6 mice exhibited a longer delivery period than tumor suppressor gene-deficient mice at the 2nd or later delivery

Aging is a nonmodifiable risk factor for infertility^35^, and we used a period of 90 days to consider aging-related reproductive decline (Fig. 2B). This experiment can allow the difference in the time between the 1st and 2nd or later delivery periods in mice to be distinguished. Surprisingly, the period between the 1st and 2nd deliveries for the wild-type mice was longer than that for tumor suppressor gene-deficient mice (Fig. 6B).

### Relationship between lifespan and perinatal mortality

A recent study proposed that both deleterious changes in early life and biomarkers of continuous aging might affect selection and define aging characteristics^9^. To understand the relationship between lifespan and perinatal mortality, we determined the lifespan of the transgenic mouse strains (Fig. 6C). Most p53^−/−^ mice died within 9 months of birth, mainly from thymic lymphoma with varying degrees of splenomegaly (Fig. 6D), and this pattern was nearly the same as that previously reported for Trp53 null (p53^−/−^) mice^27^. Consistent with the findings of a previous study^23^, APC^Min/+^ mice had a shorter lifespan than p53^−/−^ mice (Fig. 6C). In contrast, RECQL4^HD/HD^ mice exhibited nearly the same lifespan as wild-type mice (Fig. 6C), as previously mentioned^17^. However, we noticed that the slope of the Kaplan–Meier survival curve of the RECQL4^HD/HD^ mice crossed that of the wild-type mice at approximately 110 weeks, which suggested that RECQL4^HD/HD^ mice somehow have lower mortality risks associated with aging. Indeed, the cumulative mortality risk of RECQL4^HD/HD^ mice showed marked decreases with aging, particularly after 110 weeks, compared with that of wild-type mice (Fig. 6E and Table 1). Finally, we assessed the rate of food consumption in four mouse strains, because calorie restriction is a widely accepted strategy for extending the lifespan of rodents^36^. All mouse strains showed similar rates of food intake (*p* = 0.29, Fig. 6F), which suggested that the metabolic pathway had low contributions, if any, to extending lifespan of RECQL4-deficient mice. Taken together, these results suggest that RECQL4 is a pleiotropic gene and that lifespan and perinatal mortality are not correlated in such a pleiotropic tumor suppressor gene-deficient C57BL/6 mice.

**Table 1:** Age-related hazard ratios (95% confidence intervals) *RECQL4*^HD/*HD*^ mice compared with *wild-type* mice.

Age group (weeks)	Number of mice	Hazard ratio (95% CI)	*p* value
Total	Wild-type (n = 18) *RECQL4*^HD/*HD*^ (n = 17)	0.72 (0.35–1.46)	0.36
110 >	Wild-type (n = 8) *RECQL4*^HD/*HD*^ (n = 8)	2.9 (1.01–8.82)	0.047
110 <	Wild-type (n = 10) *RECQL4*^HD/*HD*^ (n = 9)	0.39 (0.15–1.07)	0.067

## Discussion

**Figure 7:**
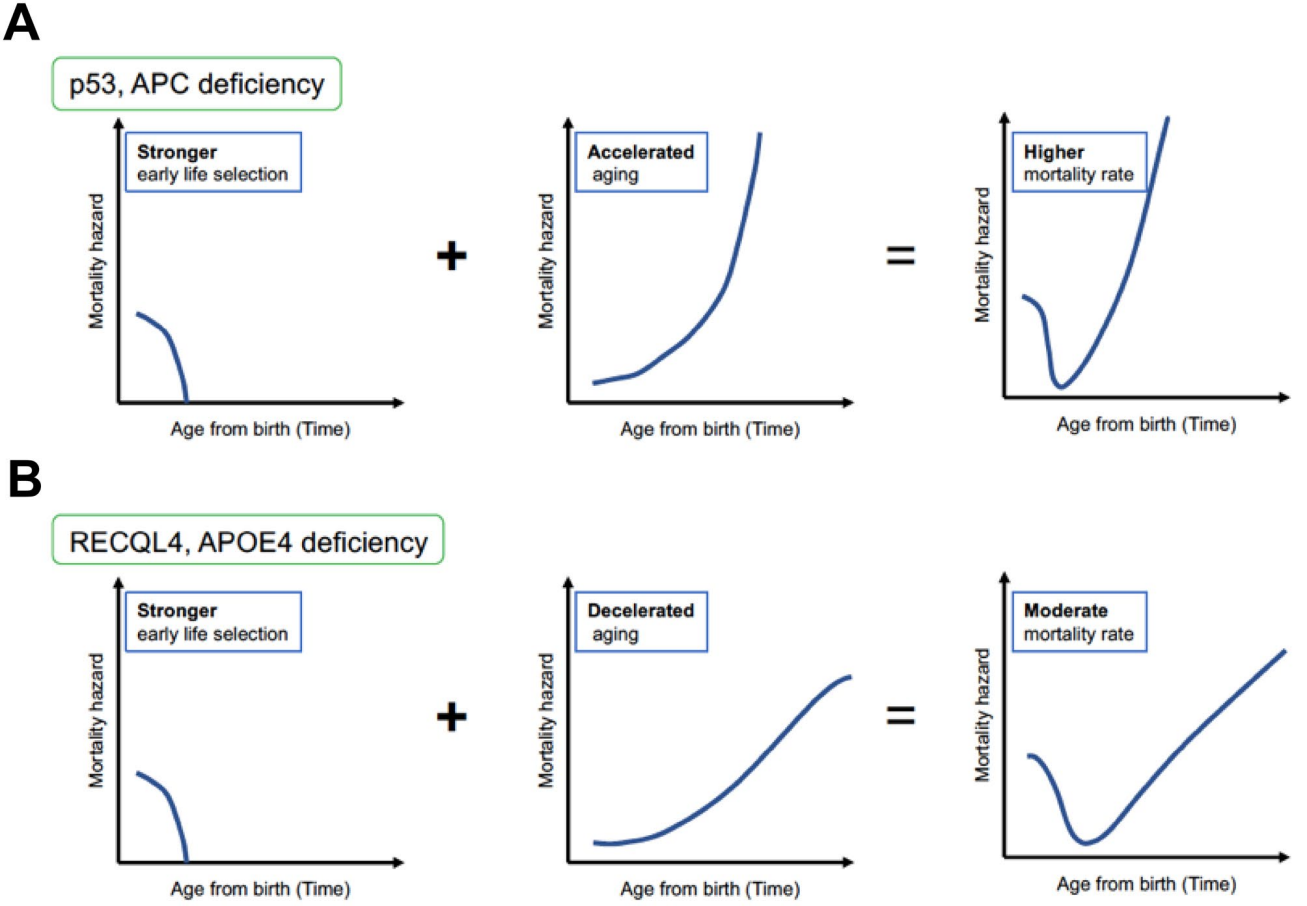
A proposed model for distinguishing between two different types of tumor suppressor gene-deficient mice to explain the difference in lifespan. (**A**) APC or p53 genes might play protective roles in early life selection and aging as expected in Fig. 1B. (**B**) Given the decrease in reproduction observed in RECQL4-deficient mice, we hypothesize that RECQL4 might exert a negative effect on aging and that the lifespan of RECQL4-deficient mice would be the outcome of the combined effect of stronger early life selection and decelerated aging. Refer to the Discussion for APOE4-deficient mice, which might exhibit a lifespan similar to that of RECQL4-deficient mice under certain environmental conditions.

Our study revealed several findings. First, p53^−/−^, APC^Min/+^, and RECQL4^HD/HD^ mice exhibited relatively stable reproduction, particularly at the 2nd or later delivery. Second, the p53^−/−^ mice showed better reproductive ability than APC^Min/+^ or RECQL4^HD/HD^ mice at the 1st delivery. Third, the p53^−/−^ mice on the C57BL/6 background in our facility did not exhibit a markedly decreased reproductive ability. Fourth, wild-type mice experienced a longer delivery period than tumor suppressor gene-deficient mice for the 2nd or later delivery. Finally, tumor suppressor genes might be related to two different types of lifespan regulation (Fig. 7), even though these genes might be associated with a similar food consumption rate (Fig. 6F).

We observed a mortality rate of 35.3% in our wild-type C57BL/6 strain (Figs. 4A, 5A), and this rate is comparable to reported data showing a mortality rate of 28.9% across hundreds of litters of C57BL/6 mice^33^. Parental cannibalism and/or desertion in the C57BL/6 strain are reasons explaining the higher mortality rate of this strain compared with that of the better breeder FVB strain^37^. We also observed a higher survival rate at the 2nd or later delivery than at the 1st delivery with the APC^Min/+^ and RECQL4^HD/HD^ mouse strains (Fig. 4E,F). Because no difference in the pup numbers just after birth was observed between the 1st delivery (Fig. 5B) and the 2nd or later delivery (Fig. 5C) among all the mouse strains, it appears unlikely that the body size of the females or the stability of hormone secretion became suitable for pregnancy at the 2nd or later delivery in the APC^Min/+^ and RECQL4^HD/HD^ mouse strains. Instead, we hypothesized that the APC^Min/+^ and RECQL4^HD/HD^ parental mice will exhibit better rearing experiences^38^ and/or might have reproductive strategies to adapt to heterogeneous environments^39^. Indeed, RECQL4^HD/HD^ parental mice showed a high frequency of absorption at an early period after the initiation of mating (*p* = 0.0018, Fig. 3D).

p53^14^, APC^31^, and RECQL4^16^ are involved in chromosomal stability. Hence, we expect that the increased perinatal deaths of pups birthed by tumor suppressor gene-deficient mice (Figs. 4, 5) resulted mainly from chromosomal instability. In fact, some developmental abnormalities, such as failure of anterior neural tube closure in p53^−/−^ mice^12^, ovarian follicle abnormalities in APC^Min/+^ mice^40^, and skeletal tissue abnormalities in RECQL4^HD/HD^ mice^17^, were observed. In contrast, the tumor suppressor gene-deficient mice showed relatively stable reproduction, with similar or even shorter delivery periods than the wild-type C57BL/6 mice (Figs. 3E, 6A,B). Genetic crossover (recombination) occurs during germline meiosis^41^. If tumor suppressor genes are involved in basal germline function, the associated transgenic mice should exhibit severe intrauterine fetal death. In contrast, tumor suppressor genes can be differentially regulated through embryogenesis^42^. Alternatively, tumor suppressor gene-deficient pups might be susceptible to postnatal physiological alterations, including alterations in respiratory function, milk digestion and absorption function, gastrointestinal motility, and body temperature control.

Activated p53 can suppress cancer but accelerate senescence, and p53 is considered one of the clearest pleiotropic genes^5^. Consistently, p53^−/−^ mice died, mainly due to cancer, during the early period (Fig. 6C), and we could not observe senescence phenotypes in the p53^−/−^ mice. Our findings regarding mild and sex-independent reproductive defects are in stark contrast with the severely decreased reproductive ability^13^ and sex differences^12^ in p53^−/−^ mice. Specifically, some RECQL4^HD/HD^ mice showed apparent abdominal distention but did not deliver pups (denoted by the diamonds in Fig. 3D), which suggested that the delivery rate was lower than the pregnancy rate. Even with these criteria, we observed a markedly higher litter size (Fig. 5A) and a notably higher delivery rate (Fig. 3E) with the p53^−/−^ mice than observed in a previous study^13^. Some studies have suggested that polymorphisms in p53 codon 72 could alter the p53 activity levels and that this alteration might affect implantation in humans and mice^43,44^. Although this polymorphism statistically affected implantation, the effect was markedly less severe than that of the implantation defect in p53^−/−^ mice^13^. In addition, p53^−/−^ mice are null for p53 expression, and the phenotype of these mice does not necessarily have to be the same as any of the phenotypes caused by p53 polymorphisms. Due to the complexity of the implantation process, a redundant mechanism could compensate for implantation failure in the absence of p53 expression^42^. We hypothesize that the phenotypic discrepancy related to the marked reduction in the reproduction of p53 mice reported by Dr. Levine’s research group^13^ compared with that of other reported p53 knockout mice^5,12,27,45,46^ might be due to differences in the experimental approaches used. Specifically, gain- or loss-of-function in some genes or noncoding regions involved in fertilization might occur during maintenance of the mouse strain. Alternatively, we favor the hypothesis that the reproductive discrepancy might be caused by environmental differences. For example, APC^Min/+^ mice were previously shown to live for approximately 6 months^23^, as observed in this study (Fig. 6C). In contrast, the same APC^Min/+^ mice on a C57BL/6 background in another laboratory lived for nearly 12 months^47^. Moreover, the behaviors and immune system of mice are easily affected by the laboratory environment^48,49^. Therefore, careful assessment of the experimental settings and analyses are needed to draw decisive conclusions about the reproductive phenotypes of transgenic mice.

Primary infertility refers to the condition of pairs who have never achieved a pregnancy, whereas secondary infertility refers to the condition of pairs who have been able to achieve at least one pregnancy. In humans, the rates of primary and secondary infertility are approximately 2% and 5%, respectively^50^, and the 1st and 2nd delivery periods might be different in mice. Notably, the period between the 1st and 2nd deliveries was longer in wild-type mice than in tumor suppressor gene-deficient mice (Fig. 6B). In mice, the length of the gestation period is strain-dependent^51^, and the different delivery periods in transgenic mice might be caused by genetic factors. The regulation of the estrus cycle and lactation by growth factors or hormones might be affected in tumor suppressor gene-deficient mice because hormone deregulation increases the risk of some hormone-related cancers, such as ovarian, testicular, and thyroid cancers^52^. However, p53, APC, and RECQL4 are involved in discrete signaling pathways [e.g., APC is involved in the WNT signaling pathway^31^, and p53 is involved in the ATM-p53-p21 pathway^30^] to suppress genome instability and tumor growth. Thus, hormone alteration in response to tumor suppressor gene deficiency appears to be a relatively unlikely explanation. The behavior of female mice (*Mus musculus*) is affected by the sex composition of the litter, and female mice can recognize the sex of their littermates^53^. Alternatively, but not exclusively, we may explain the reason for the shorter period between the 1st and 2nd deliveries in three independent tumor suppressor gene-deficient mice strains (Fig. 6B) by the trade-off between fecundity and aging (Supplemental Fig. 2). The limitation of this study is the lack of mechanistic data, and mechanistic insights at the molecular and cellular levels will help us understand the relationships among fecundity, cancer and aging in rodents. In addition, we used mainly C57BL/6 N mice as a wild-type control except survival assay (Fig. 6C, E) for comparison among tumor suppressor gene-deficient mice, which were established either in C57BL/6 N or C57BL/6 J background. This point should be taken into account for future experiments to obtain clearer results. Further investigation with larger sample numbers is needed to determine the correlation between the delivery period and the pup numbers (survival rates) at weaning in C57BL/6 mice from physiological perspectives.

Consistent with the results of a previous study^17^, RECQL4^HD/HD^ mice had a normal lifespan (Fig. 6C), although higher perinatal mortality was observed in RECQL4^HD/HD^ mice that in wild-type and p53^−/−^ mice (Fig. 4D,E). Notably, we observed a higher abortion rate in RECLQ4-deficient mice than in the other mouse strains (Fig. 3G). This result suggests loss of an embryo with chromosomal abnormalities and/or highly damaging mutations in RECQL4^HD/HD^ mice at mid- to late-gestation^54^, even though RECQL4-deficient mice exhibited a lower mortality risk with aging (Fig. 6E, Table 1). The possibility that such a higher negative selection at mid- to late-gestation against parental deletion alleles among RECQL4^HD/HD^ mice might result in increased survival with age as a trade-off relationship cannot be ruled out. If a recent aging hypothesis proposes that deleterious changes in early life might determine aging and lifespan is correct^9^, RECQL4 deficiency should positively affect viability later in life (Fig. 7B), similar to the pleiotropic APOE4 allele^55^, which can produce high fertility and protect against perinatal death^56,57^ but can increase the later risk of Alzheimer’s disease in the elderly population^58^. Indeed, dysregulation of RECQL4 is associated with several human cancers^59^. Moreover, a recent study proposed that mice with RECQL4 truncating mutations do not exhibit tumorigenesis even after exposure to ionizing radiation^60^, which suggested that RECQL4-deficiency might play an important role in protecting against deleterious effects later in life in mammals (Fig. 7B). Further studies are needed to determine whether this effect is also observed in other RECQL4-deficient mice or patients with RTS.

The human conditions LFS, FAP, and RTS correspond to the phenotypes of p53^−/−^, APC^Min/+^, and RECQL4^HD/HD^ mice, respectively. However, the knowledge about fertility compromise in patients with LFS and RTS is limited because patients with LFS exhibit a high incidence of cancer at younger ages than in the general population^26^, and the number of patients recognized to have RTS worldwide is only in the hundreds^21^. Regarding fertility in patients with FAP, a study suggested no association between the FAP phenotype and fertility^61^. Importantly, the reproductive phenotypes in humans with singleton pregnancies might be distinct from those of litter-bearing (multiparous) mice. Whether other tumor suppressor gene-deficient mice show similar phenotypes under the same experimental conditions remains to be determined before generalization of the reproductive ability of tumor suppressor gene-deficient humans and mice.

## Conclusion

Our findings suggest that tumor suppressor gene-deficient mice exhibit relatively stable reproduction, with an increased risk of perinatal mortality. Moreover, we revealed an age-dependent trend in which wild-type paired mice exhibited a longer delivery period than tumor suppressor gene-deficient mice, regardless of the genotype, which might reflect the trade-off between fecundity and aging^9^; the only exception was that RECQL4 mice showed a pleiotropic phenotype. The simple experiments used in this study combined with the Kaplan–Meier method and Cox regression analyses might provide phenotypic analyses that can allow researchers to understand the relationship among early life selection, carcinogenesis, and senescence.

## Materials and methods

### Mice

p53^−/−^ mice were originally obtained from Dr. Y. Gondo^45^ and maintained at our facility with more than ten backcrosses with the C57BL/6 N strain (more than 99.9% identical to the C57BL/6 N background)^62^. APC^Min/+^ mice were purchased from The Jackson Laboratory (no: 002020) and maintained at our facility with more than eight backcrosses with the C57BL/6 J strain (more than 99% identical to the C57BL/6 J background). RECQL4^HD/HD^ mice were obtained from Dr. V.A. Bohr^63^ and maintained at our facility with more than six backcrosses with the C57BL/6 J strain (more than 98% identical to the C57BL/6 J background)^64^. We used the C57BL/6 J from Japan Charles River for backcrosses because the Jackson Laboratory introduced C57BL/6 J Mice to Japan Charles River in F218 in 2002, and the C57BL/6 J mice maintained by Japan Charles River are C57BL/6 J from The Jackson Laboratory. These three tumor suppressor gene-deficient mouse strains were more than 98% identical to the C57BL/6 background, and C57BL/6 N mice were purchased from Japan Charles River Laboratories Inc. (Yokohama, Japan) and served as a parental wild-type control mouse for most experiments. C57BL/6 J mice were purchased from Japan Charles River Laboratories Inc. (Yokohama, Japan) and served as a parental wild-type control mouse for survival assay. The mice were housed in rooms with controlled temperature (22 ± 1.5 °C) and humidity (50 ± 10%) and a standard 12-h light/12-h dark cycle in a specific pathogen-free environment. The mice were maintained on sterile water and a standard pellet diet (MF, Oriental Yeast Co., Ltd., Japan). All care and use of the animal subjects followed the guidelines of the ARRIVE guidelines, Laboratory Animal Research Center and the Use Committee of the University of Occupational and Environmental Health, Japan, and the protocol was approved by the Committee on the Ethics of Animal Experiments (Permit Number: AE14-026, AE15-015, 016).

### Evaluation of reproductive parameters using a breeding assay

Because litter size is a representative reproductive trait and is influenced by genetic or environmental changes^65^, we focused on the litter size of four different mouse strains. Four-week-old mice were weaned from parental mice, and mating was initiated at 8 weeks using a randomly determined mating pair (Fig. 2B). The mating pairs were kept in cages for at least 90 days. Some APC^Min/+^ or p53^−/−^ mice died during the 90-day holding period (denoted by crosses in Fig. 3). We observed the mice almost daily, particularly near the delivery period. The pup numbers and survival rates 3 weeks after birth were examined to evaluate the stable postnatal period. The pup numbers just after birth were examined to evaluate the perinatal reproduction period. The delivery rate was calculated as the ratio of the number of females with confirmed delivery to the number of female mice housed with male mice. At 4 weeks of age, the delivered pups were relocated to new cages to prevent mating with the parent mice (Fig. 2B). At this point, we confirmed which sex of pups can survive at weaning. The total numbers of breeding pairs for wild-type, p53^−/−^, APC^Min/+^, and RECQL4^HD/HD^ mice were 14, 12, 9, and 9, respectively (Fig. 3). Among these breeding pairs, the number of 1st deliveries obtained with the wild-type, p53^−/−^, APC^Min/+^, RECQL4^HD/HD^ mice was 11, 11, 8, and 8, respectively (Supplemental Fig. 2). In addition, the number of 2nd or later deliveries obtained with the wild-type, p53^−/−^, APC^Min/+^, and RECQL4^HD/HD^ mice was 12, 20, 11, and 8, respectively, during the 90-day period (Supplemental Fig. 2).

As an independent experiment, some female mice were weaned at 6 weeks of age to evaluate whether they exhibited early sexual maturity to support pregnancy.

### Histological analysis valuation of the rate of food int

The excised mouse tissues were fixed in 10% neutral-buffered formalin, dehydrated, and embedded in paraffin by conventional methods. Subsequently, 5-mm sections were cut from formalin-fixed paraffin-embedded blocks and transferred to glass slides. The sections were stained with H&E using standard procedures, and images were obtained with an BX-53 microscope (OLYMPUS, Tokyo).

### Evaluation of the rate of food intake

For wild-type and p53^−/−^ mice, two cages of male mice and two cages of female mice (a total of four cages) were examined, whereas for APC^Min/+^ and RECQL4^HD/HD^ mice, three cages of male mice and two cages of female mice (a total of five cages) were examined. We weighed the food given to the mice daily and divided the weight of the reduction in food each day by the number of mice and hours to obtain the rate of food intake (gram/hour).

### Statistical analysis and reproducibility

All statistical tests were two-sided. All the analyses were performed using GraphPad Prism (version 6.0 g). For two-group comparisons, the data were analyzed by a Kolmogorov–Smirnov test to assess the normality of the distribution. We rejected the null hypothesis if *p* < 0.05. Normally distributed data were analyzed using Welch’s t-test. Data with a nonnormal distribution were analyzed using the Mann–Whitney U test. In the Kaplan–Meier survival analysis, *p* values were determined using the Gehan-Breslow-Wilcoxon test. For analysis of the abortion rate and the rate of food intake (g/h), we used ordinary one-way ANOVA for multiple comparisons among the four strains. For the analysis of sex bias, we used the nonparametric Wilcoxon matched-pairs signed-rank test. A Cox regression analysis was used to estimate the hazard ratios of the RECQL4^HD/HD^ mice compared with the wild-type mice with R-4.0.3. No statistical methods were used to predetermine the sample size.

## Supplementary Information

Supplementary Information.
