# Supplementary material for: Comparison of the fertility of tumor suppressor gene-deficient C57BL/6 mouse strains reveals stable reproductive aging and novel pleiotropic gene
Source: Sci Rep. 2021 Jun 11;11:12357. doi: 10.1038/s41598-021-91342-9 (PMC8195996; doi:10.1038/s41598-021-91342-9)
Supplement: Supplementary file 1 — Supplementary Information. [file 41598_2021_91342_MOESM1_ESM.pdf]

Title page

Comparison of the fertility of tumor suppressor gene-deficient C57BL/6 mouse strains reveals stable reproductive aging and novel pleiotropic gene

Authors

Masaoki Kohzaki[1*], Akira Ootsuyama[2], Toshiyuki Umata[3] and Ryuji Okazaki[1]

Affiliations

1. Department of Radiobiology and Hygiene Management, Institute of Industrial Ecological Sciences, University of Occupational and Environmental Health, Japan, 1-1 Iseigaoka Yahatanishi-ku, Kitakyushu, 807-8555, Japan.

2. Department of Radiation Biology and Health, School of Medicine, University of Occupational and Environmental Health, Japan, 1-1 Iseigaoka Yahatanishi-ku, Kitakyushu, 807-8555, Japan.

3. Radioisotope Research Center, Facility for Education and Research Support, University of Occupational and Environmental Health, Japan, 1-1 Iseigaoka Yahatanishi-ku, Kitakyushu, 807-8555, Japan.

***Corresponding author:**

Masaoki Kohzaki, Ph.D. (https://orcid.org/0000-0002-5020-8497)

Iseigaoka Yahatanishi-ku, Kitakyushu, 807-8555, Japan. Tel: +81 93 691 7549, Email: masaoki-k@med.uoeh-u.ac.jp

Title: Comparison of the fertility of tumor suppressor gene-deficient C57BL/6 mouse strains reveals stable reproductive aging and novel pleiotropic gene

Supplementary Figure 1

A

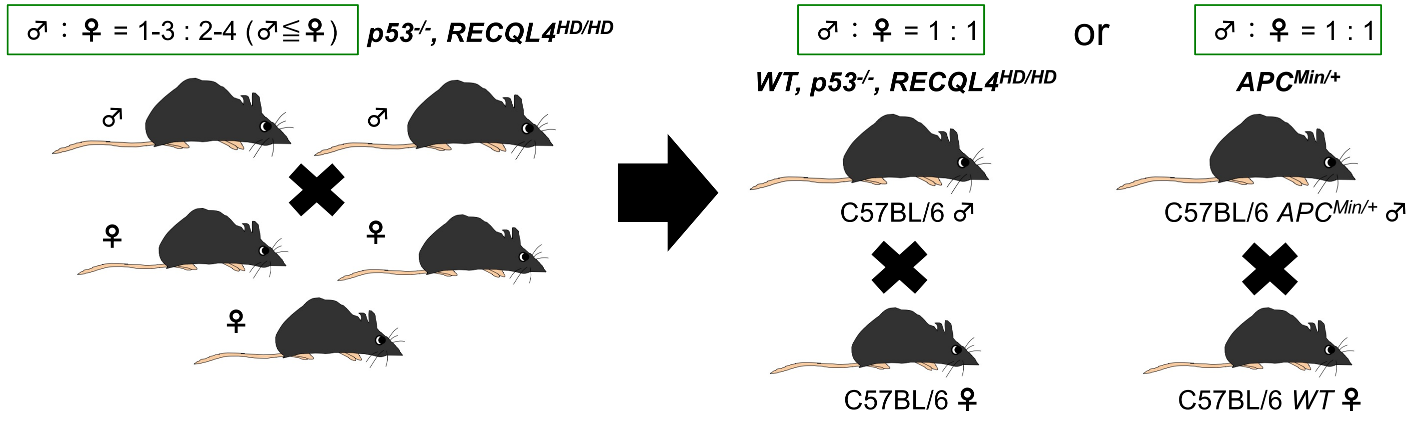

B

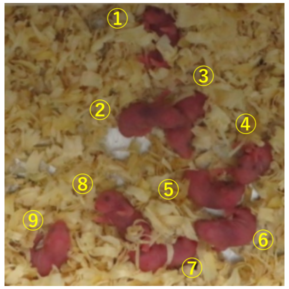

Nine $p53^{-/-}$ pups

(A) For less-reproductive mouse strains, multiple pairs were used to maintain the strain.

(B) Representative image of many p53-/- pups. Nine pups (circles with numbers) were recognized in this case.

Title: Comparison of the fertility of tumor suppressor gene-deficient C57BL/6 mouse strains reveals stable reproductive aging and novel pleiotropic gene

Supplementary Figure 2

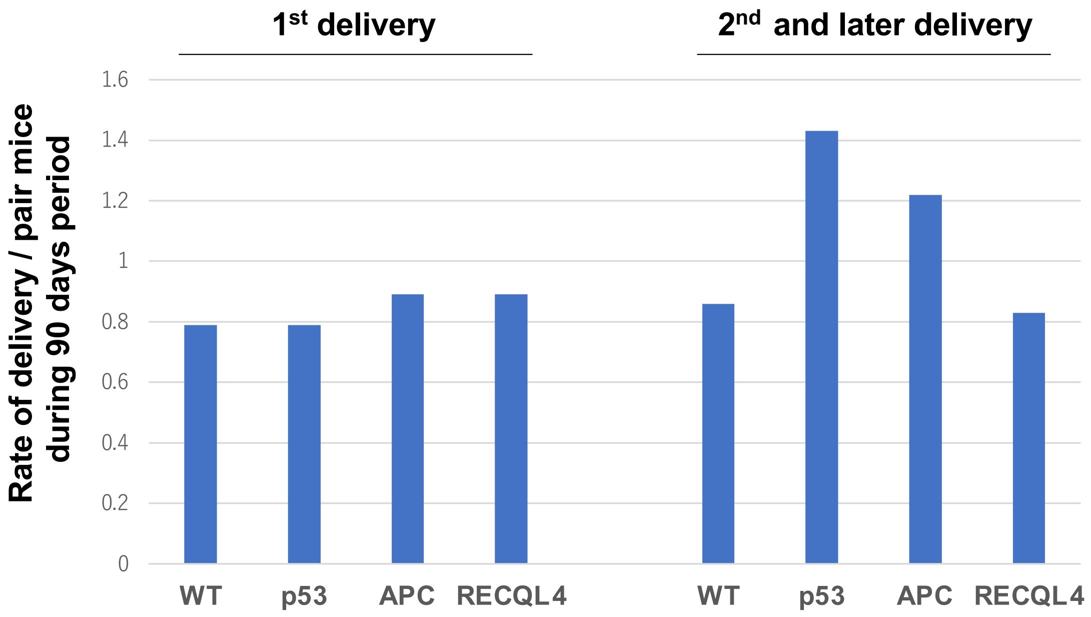

The rate of delivery was calculated by the number of delivery at either 1st delivery or 2nd and later delivery, divided by the total number of breeding pairs for wild-type, $p53^{-/-}$, $APC^{Min/+}$, $RECQL4^{HD/HD}$ mice
